# Understanding the informal aspects of medication processes to maintain patient safety in hospitals: a sociotechnical ethnographic study in paediatric units

**DOI:** 10.1080/00140139.2024.2333396

**Published:** 2024-04-01

**Authors:** Adam B. Sutherland, Denham L. Phipps, Suzanne Grant, Joanne Hughes, Stephen Tomlin, Darren M. Ashcroft

**Affiliations:** a Medicines Optimisation Research Group, School of Pharmacy & Medical Sciences, Faculty of Life Sciences, University of Bradford, Bradford, UK; b Division of Pharmacy & Optometry, School of Health Sciences, Faculty of Biology, Medicine & Health, University of Manchester, Manchester, UK; c NIHR Greater Manchester Patient Safety Research Collaboration, Manchester, UK; d Pharmacy Department, Royal Manchester Children’s Hospital, Manchester University NHS Foundation Trust, Manchester, UK; e Division of Population Health and Genomics, School of Medicine, University of Dundee, Dundee, UK; f Mother’s Instinct, Cambridge, UK; g Children’s Medicines Research & Innovation Centre, Great Ormond Street Hospital NHS Foundation Trust, London, UK

**Keywords:** Paediatrics, medication safety, human factors, systems ergonomics, ethnography

## Abstract

Adverse drug events (ADEs) are common in hospitals, affecting one in six child in-patients. Medication processes are complex systems. This study aimed to explore the work-as-done of medication safety in three English paediatric units using direct observation and semi-structured interviews. We found that a combination of the physical environment, traditional work systems and team norms were among the systemic barriers to medicines safety. The layout of wards discouraged teamworking and reinforced professional boundaries. Workspaces were inadequate, and interruptions were uncontrollable. A less experienced workforce undertook prescribing and verification while more experienced nurses undertook administration. Guidelines were inadequate, with actors muddling through together. Formal controls against ADEs included checking (of prescriptions and administration) and barcode administration systems, but these did not integrate into workflows. Families played an important part in the safe administration of medication and provision of information about their children but were isolated from other parts of the system.

## PRACTITIONER SUMMARY

Formal medicines safety processes in paediatric units are disjointed and disconnected. This has led actors in the system (e.g. nursing and medical staff) to develop informal adaptations to increase resilience. There is a need to incorporate these adaptations into a systems-focussed consideration of safety processes, in order to properly inform the development of medication safety interventions.

## Introduction

All healthcare carries potential for harm and underlying risks are often considered in the context of preventability. Recent estimates suggest that up to 25% of preventable patient safety incidents are associated with medication, of which 6% result in severe harm or death (Panagioti et al. 2019). The World Health Organisation has set a target to reduce the incidence of preventable medicines-related harm by 50% (Donaldson et al. 2017). In England it is estimated that there are around 237 million medication errors every year, of which 28% (∼66 million) are associated with harm. The latter cost the National Health Service (NHS) almost £100 m per annum, and may contribute to around 1700 deaths (Elliott et al. 2021).

Children appear to be more at risk from medication errors than other types of patient (Kaushal et al. 2001). This may be partly because of the need for bespoke doses based on their weight, as well as the common practice of using unlicensed and off-label medication (that is, using medicines that are not approved by regulators for use in children and infants) (Benn 2014). Recent data have estimated that in paediatric settings, between 1 and 2.6% of medication errors are associated with some degree of harm, although more than 9 in 10 of these were of minor severity (Gates et al. 2018).

Most studies have focussed only on prescribing errors; however, medication use is a complex process of multiple interconnected tasks and operators. Practitioners need to adapt their practice in order to accommodate the variation that these interactions between system components introduce, resulting in a complex adaptive system (Reason 1990; Catchpole and Jeffcott 2017). In addition to prescribing, other key processes include dispensing, preparation, administration and monitoring or follow up (Walsh, Kaushal, and Chessare 2005). It appears that most preventable patient safety incidents involving paediatric medicines are related to discrepancies in medication documentation on admission or discharge from hospital, or to administration of medicines (Sutherland, Phipps, et al. 2019).

Existing empirical research has focussed largely on the characterisation of the prevalence and nature of patient safety incidents in broad population groups, from which causative and contributory factors have been inferred (Sutherland and Phipps 2020). The causative factors of these incidents have been identified through a comparison to an idealised ‘standard’ of work that is described in rules, policies and procedures. Viewing work and the outcomes of work through this lens is termed ‘work as imagined’. (WAI)(Hollnagel 2015) WAI assumes that work processes are linear and can be completed as intended under all conditions. However, in complex adaptive environments such as healthcare, operators have to adapt and adjust their work to account for variation in events and outcomes caused by the unpredictability of all parts of the system (Read et al. 2021). Consequently, the development of interventions through a WAI lens to ‘improve’ medication safety have been largely ineffective because they do not take account of this adaptive human behaviour described as ‘work-as-done’ (WAD).

There is little research in medication safety that has taken the system-focused perspective that is characteristic of ergonomics and human factors (HF/E). Carayon et al., amongst others, have demonstrated that taking such a whole-system perspective in which human performance is viewed in the context of the interaction between people, tools, tasks and the organisational and physical environment can generate a rich, in-depth understanding of healthcare work as a complex adaptive system which is potentially useful in improving the safety of medication processes as they are done in practice (Carayon 2012; Carayon et al. 2014). So, in order to better inform work design in healthcare settings, there is a need to understand ‘Work-as-Done’ from a whole system perspective. This paper presents findings from a large exploratory study designed to answer questions about the systems-related contributory factors to drug-related problems and potential ADEs in hospitalised children and young people. In this paper we explore the everyday informal ‘work as done’ in medicines management in paediatric in-patient units using a systems-focused approach.

## Method

### Research setting

The study was conducted across three acute children’s wards in the North of England between October 2020 and May 2022 using a multi-site ethnographic design. The three study sites were purposively sampled to represent a diversity of organisational structures (i.e. a standalone specialist hospital; specialist hospital forming part of a larger hospital group; district general hospital), populations served (in terms of social, cultural and economic diversity) and geographical location (Table 1).

**Table 1.** Characteristics, location and size of study sites.

	Location	Hospital	Unit size
GH1	A small town in the northwest of England; pop. 55000	District general (245 beds). Neonatal closed prior to initiation	12 beds, and a six-bed assessment area
CH1	A post-industrial city on the north-west coast of England; pop. 500,000	Standalone tertiary children’s (270 beds). Neonatal care provided off-site	28 beds
CH2	Medium-sized city in northern England; pop. 800,000	The children’s hospital (286 beds) on a city centre hospital site (1100 beds); part of a multi-hospital trust (2500 beds)	12 beds. Other secondary care admissions were distributed elsewhere in specialist areas based on bed availability.

### Data collection

Ethnographic observations were undertaken using the focussed ethnographic method that involved studying a defined problem using non-participant observation methods, over a relatively short period of time (<90 hours per site), with a single researcher who was independent of the field (Andreassen, Christensen, and Møller 2020; Bikker et al. 2017). The observational data was supported by in-depth interviews with participants. The researcher (AS) was a male doctoral student in their mid-40s. He was a qualified paediatric pharmacist of over 20 years standing working in paediatric critical care at a non-participating hospital. He adopted appropriate working attire to blend in with the wider clinical team.

A total of 230 hours of non-participant observation was undertaken between October 2020 and May 2022 with 404 observation participants. At each site, everyday activity conducted by healthcare staff and families was observed, with a focus on all medicines-related activities (i.e. prescribing, preparation, administration and monitoring). All participants were asked for their consent immediately prior to observation. Ward rounds, handovers, medication rounds and pharmacy service rounds were attended and observed as and when they occurred. Discreet observation of participant activity was undertaken both stood at the nurses’ station and moving around the ward. During observations, informal interviews were conducted with participants to clarify or elaborate on what was being observed (Shorrock 2022; Standing and Tuleu 2005).

Semi-structured interviews were carried out to provide complementary data to the observations and identify activities or events requiring observation (Becker and Geer 1970; Atkinson and Coffey 2003; Charmaz and Belgrave 2012; Lofland et al. 2006). 19 interviews were conducted, with participants identified using a maximal variation purposive sampling strategy (Hammersley 2002; Miles and Huberman 1994). The sample was designed to cover diverse experience in the prescribing, preparation and dispensing or administration of medicines. Organisational perspectives were also considered important, the experiences of Medicines Safety Officers (MSO) were also sought. These participants represented the organisational perspective on medicines safety, having accountability for this aspect of care quality within NHS organisations. (Table 2)

**Table 2.** Purposive sample and characteristics of interview participants.

Participant ID	Role	Location
1	Pharmacist (Pilot Interview)	Pilot site (not included in analysis)
2	Nurse	CH1
3	Pharmacist	GH1
4	Nurse	GH1
5	Junior Doctor	GH1
6	Pharmacist	CH1
7	Consultant	CH1
8	Consultant	GH1
9	Junior Doctor	GH1
10	Medication Safety Officer (Nurse)	CH1
11	Medication Safety Officer (Pharmacist)	CH1
12	Pharmacist	CH2
13	Pharmacist	CH2
14	Medication Safety Officer (Nurse)	CH2
15	Parent	CH2
16	Nurse	CH2
17	Parent	CH2
18	Junior Doctor	CH2
19	Parent	CH1

Interview participants were recruited using the purposive approach described in Table 2. Potential participants were identified based on their involvement with medicines and medicines safety processes in the participating organisation, and their lived experience of medicines safety. The sample included nurses, medical staff (consultants and trainees), pharmacists, medicines safety officers, parents, and carers. The interview topic guide was adapted from a previous study on the management of acute kidney injury (Phipps et al. 2019). This topic guide is available in Supplementary File 1, and explored participants’ understandings of the medication safety systems in their place of work, their perceptions of the work of medication safety and their experiences within those systems. With permission, all interviews were audio recorded and transcribed verbatim for analysis. The study was approved by the Health Research Authority Leeds West Research Ethics Committee (19-YH-0430).

### Data analysis

Fieldnotes were maintained using a secure tablet computer and were transcribed into observation narratives as soon as possible after observation. Reflective comments and questions were also incorporated into these narratives. Field notes and interview transcripts were anonymised, collated, and managed using qualitative data management software (NVivo v12, QSR International). Coding was undertaken in duplicate by the lead researcher and a data analysis team consisting of the researcher (AS), a HF/E expert (DLP), a social anthropologist (SG) and a patient representative (JH). An open coding approach using Braun and Clarke’s thematic analysis framework was used (Braun and Clarke 2006). Familiarisation with the data was supported through the conversion of field notes into rich narrative records, while interview transcripts were read and re-read against the recordings for accuracy and completeness.

Codes, themes and categories were defined and agreed by consensus. Members of the data analysis team were provided with data and encouraged to read and re-read them as part of their coding. Questions and reflective thoughts were captured through a reflexive diary for the researcher and through conversation among the analytical group. Preliminary themes were identified through scrutiny of the fieldnotes and interview transcripts and a coding framework was developed that was embedded in the data collected. Codes were then organised into overall themes, which supported sense-making from the data. Deductive and inductive analytical approaches were employed during this process, with sociotechnical themes – environment, tasks, people and teams, tools and equipment, and organisational factors used as sensitising concepts during the analysis process (Holden et al. 2013). This guided thematic abstraction and the identification of the overall insights in medicines safety practices, which were then labelled among the group. (Corbin and Strauss 2016). This constant comparative approach continued until no further categories emerged.

## Results

The purpose of this study was to explore the reality of work related to medicines and safety in three busy children’s wards, to understand how work-as-done was manifested in order to derive systems-related insights into how safety was maintained in busy children’s wards. We have presented an initial description of the ‘work as done’ and then related that to the findings using the themes and categories identified in the analysis. Finally, we offer an exploration of the performance influencing factors that have emerged through this organisation of the data. We have offered quotes and fieldnote extracts throughout which are presented thus:

(Interview XX, Participant Designation (Nurse, Junior Doctor, Consultant, Pharmacist, Parent))

(Fieldnote, Activity/Observation, Site)

### Work as done

Three core elements of medication safety processes were identified – the physical space, the situational space and the cognitive aspects of medicines safety. These are illustrated (including their relationship with sociotechnical categories) in Table 3.

**Table 3.** Analytical summary and relationship with sociotechnical categories.

Inductive theme	Deductive Socio-technical Categories	Inductive Analytical Categories
Physical Space	Tools & Equipment Environment Tasks	Workstations and spaces Storage and access Distractions and Interruptions
Situational Space	People & Teams	Relationships Communication Parental involvement
Cognitive space	Organisational Factors Tasks	Conflicting priorities Knowledge and experience

#### The physical space

**Figure 1.**
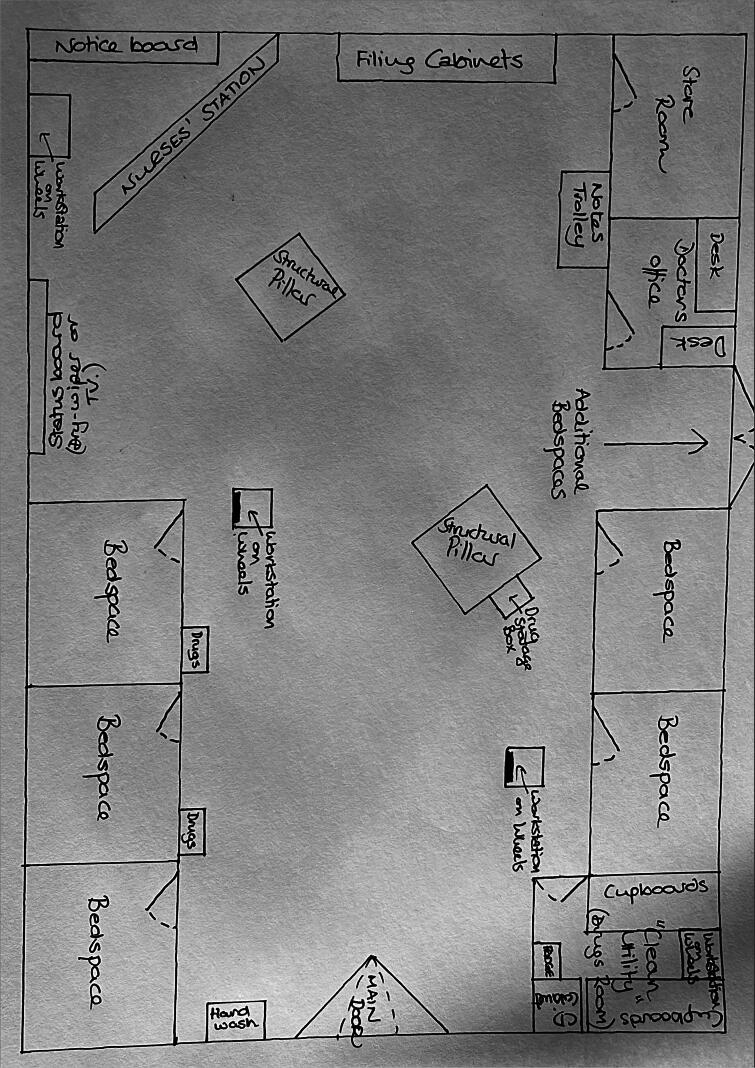
General representation of a paediatric ward.

Children were cared for in dedicated wards, with a parent accommodated with them. The layout of the spaces across the fieldsites was similar despite physical differences in size and location, and a generalised representation of a ward is presented in Figure 1.

##### Workstations and spaces

Medication was supposed to be stored and prepared in designated areas of the ward. The central medicines storage space was a ‘clean utility’. This was usually a small room on the ward, that had multiple uses and was not dedicated to medicines alone also being used to store dressings, feeds and other equipment. In two sites there were electronic prescribing systems in place, but neither clean utility contained adequate computer facilities. In CH1 a mobile computer on a large trolley (a workstation-on-wheels (WoW)) was permanently set up in the clean utility, which took up the space of one person, resulting in overcrowding when two nurses were working together.

##### Storage and access

These rooms were also secure to protect medicines from theft and misappropriation. ‘Has anyone got the keys?’ was a frequently heard announcement in all wards as nursing staff tried to get access to medicines. In conversation, all staff were able to relay stories of delayed medication because they were unable to access medicines. Nursing staff associated the keys with symbolic power and significance around the security of the medicines in their charge and bore their responsibility for medicines security heavily.

Yeah, the cold sweat when the work office calls you after a nightshift and you can suddenly feel the bunch of keys in your pocket that you’ve had on you since the 6am medication round … (Fieldnote, Observation discussion, Nurse, GH1)

An adaptation to this problem was to store medicines in other places. As well as sanctioned solutions such as lockers in patient bedspaces, and trolleys near nursing workspaces, it was common to observe medicines stored informally, or nursing staff carrying a few plastic ampoules of saline or salbutamol for nebulisation in their uniform pockets.

A pack of ipratropium and salbutamol nebulisation ampoules are left on the nurse’s station and when asked why they’re there the nurse at the desk advises that they’ll go through them all in a shift, and there’s someone there all the time. (Fieldnote, Observation discussion, Nurse, CH2)

Most medicines preparation however was observed to occur at the nurses’ station. Clean utilities were observed to be too small for the number of people required to manipulate and prepare medicines appropriately, and often lacked access to computer facilities that are essential in modern IT-centric services.

##### Distractions and interruptions

Within all sites, distractions and interruptions were everywhere in the physical and social environments – device alarms, inoperable equipment, doorbells, parents and other professionals asking for information or help. All participating sites had a ward clerk or receptionist, but these staff members were usually part time, or only deployed during normal business hours. At all other times it was usually the nurses who would answer the phone or the door. In CH1 the ward clerks worked in an office at the back of the ward, away from the front door, and it was the nurse in charge for the shift who would usually man the front door. Usually, this nurse was not directly caring for children, and was able to manage this source of distraction,

 … but when we’re in the numbers [allocated patients] … well, it’s anyone’s game really. (Fieldnotes, Nursing observation, CH1)

All professionals involved in the study believed that ‘distractions’ were a major cause of medication errors, but management of them fell to individual judgement. In conversation with pharmacists and nursing staff, the perception was that all interruptions were bad and to be avoided, but there was an acknowledgement of the reality of busy clinical life. It was likely that distractions and interruptions were unavoidable, and subsequently the interventions deployed inappropriate.

Someone needs something off you and they’re going to interrupt you whether you want them to or not …  (Interview 2, Nurse)

Staff within the environment were also isolated. As illustrated in Figure 1 there was a nurses’ station, in some wards there was also a doctors’ office and in one site there was also a pharmacy office. In all sites, patients and families were cared for in single rooms or isolation bays with limited visiting. While there was a strong social component observed to the way work was conducted, there was little sharing of space to accomplish the same task between different professionals. In the site without an identifiable doctors’ accommodation on the ward, it was seen that medical staff were mobile and would deal with problems as they emerged on a priority basis.

Post-take ward round, the nightshift doctors look exhausted. […] The consultant asks them about a medication history for the patient we are currently seeing. ‘To be honest we have just been firefighting all night, and haven’t had time to use the toilet, let alone write out a list of drugs … ‘ The round agree that pharmacy will pick the drug history up. (Fieldnotes, Medical observation, CH1)

#### The situational space

Situational spaces were places where people interacted with other people, the environment and other sociotechnical aspects of the system.

##### Teamwork

Medical staff worked in diffuse teams with responsibility across multiple different areas. Medical staff were allocated patients based on their skills and experience but would often be encouraged to volunteer to look after children that they could learn from.

Observing a handover ward round. A child with a rare disease has been admitted. One of the more senior doctors on the handover round describes their experience of this condition and a colleague announces they would like to manage them this evening. (Fieldnote, Observation, CH1)

Allocated patients were sometimes cared for in disparate parts of the hospital estate, which resulted in a divided focus. Medical staff would be reviewing a patient in the participating ward, but then be contacted and asked for advice about a patient in a different part of the hospital.

Nursing staff were employed directly as part of the ward infrastructure and would look after only patients on the ward. While each nurse was allocated several patients based on an arithmetic ratio (between 4 and 8 patients to one nurse throughout the study) they would also be asked to look after patients through the rest breaks of other nurses.

Pharmacy staff were allocated to a given ward for a short period each day – usually two or three hours. This period was unpredictable, often uncommunicated to the ward staff, and was occasionally withdrawn to cope with wider service demands in the pharmacy itself. Pharmacy staff focussed on logistics of medicines supply, and verification of medication histories either at admission or on discharge.

Families meanwhile were usually in attendance throughout their child’s stay in hospital. Formally recognised family tasks within the medicines management system included consent to treatment and providing feedback on However they were also observed providing essential pharmaceutical information about their medication and supporting medication administration. These activities were not formally recognised but were an essential part of the system.

Because if the parents are doing it, it’s one thing less for me to worry about … they know what they’re doing because they do this every day at home. (Fieldnote, Observation discussion, Nurse, CH2)

Coming into hospital was disorientating for families and often disruptive of their everyday routines. Hospital routines in most sites were described by nurses as being ‘patient-centred’ but the experience of families was that this was superficial and medication administration particularly reverted to system-centric schedules. The system centric schedules for medicines were often related to the time when the orders were entered into the prescribing system (be it electronic or on paper) as prescribers would seldom adjust times to meet patient routines.

Every time we come in here it all falls apart. I know that schedules and routines are different here, but if he doesn’t get his nitrazepam on time he gets dystonic and it just makes everything worse … (Fieldnotes, Parent observation, CH1)

Similarly, the expectations the hospitals held of parents were unclear. Parents were often observed continuing to give medicines until they were instructed to stop.

In all sites, most medicines required ‘independent second checks’. Which involved two nurses independently verifying the preparation of a medicine against a prescription. However, very few checks were completed. Nursing staff would work co-operatively talking each other through their process, in what was described as a ‘primed check’

… people show you the bottle and go, that’s paracetamol, I want 3ml and the other person just says, yes or no. (Interview 02, Nurse)

Pragmatic lists of single-check items were available but the selection of these items was perceived by nursing staff to be illogical and unpredictable, but they would just accept those idiosyncrasies.

Salbutamol inhalers are absolutely fine to give single check, but the minute you put a yellow spacer on it … double check. (Fieldnote, Observation discussion, Nurse, CH2)

#### Communication

All sites had developed quasi-formal mechanisms of capturing and transferring information about patients quickly and succinctly. In all sites there were printed documents that captured information about each patient in a standardised format that were referred to as ‘The Handover’. There were different Handovers for medical, nursing and pharmacy staff and each document captured different information. They were maintained independently by their users and were updated on-the-go as things changed, when decisions were made, or at fixed time points in the day alongside other formal processes like ward rounds. Almost everyone carried a paper copy of ‘The Handover’ with them, and used them as a record of tasks achieved, details and jobs that required attention, or as a plan for the shift. Nursing staff would often show colleagues their Handovers to show how busy they were. A two-page Handover document was often considered emblematic of a busy shift.

#### The cognitive space

Our findings around the cognitive aspects of medicines use and safety focussed on the training and expertise of the actors in the system and how they used this expertise to accomplish their tasks.

#### Priorities and objectives

In all three fieldwork settings, actors in the system worked separately, attending to their own objectives until a problem or question emerged that required collaboration. Multi-professional working was not routine. Medical ward rounds consisted solely of medical team members and medical students and were used as the primary decision-making vehicle for the care of the patient. Ward rounds were conducted with the medical notes, and a physical examination of the patient and discussion with their resident parent or carer. Ward rounds were centred on a trolley, either with a computer or carrying physical paper notes. The consultant would lead the round, with a trainee doctor writing up notes and plans. Parents would be asked for their concerns and impressions of progress, but decision making was medically led, focussed on the primary problem that resulted in admission to hospital.

Nursing work was focussed on the nurses’ station with them articulating their medicines work with other tasks and duties. They synthesised their activities from the documented medical plan, and verbal interaction with the medical team and families. Parents and families were not viewed by professionals as having a formal role in this system but were observed being co-opted to support medication administration and information co-ordination.

The pharmacy team were not part of the ward hierarchy and thus were not a part of the routine handovers or decision-making processes. Pharmacy teams were never observed to be part of a ward round, and all contact with clinical teams around medication issues was in response to identification of a problem. This reactive role created tension between pharmacy teams and colleagues in other fields.

When approached by a pharmacy professional with a question, medical staff were observed to joke ‘Oh god, what have I done now?’ (Fieldnotes, observation, CH2)

#### Medicines decision making

Normal prescribing tasks were undertaken alone until either a confirmatory check or additional information was required. Medicines were prescribed according to manuals (e.g. the British National Formulary for Children) or guidelines which were all intended to be accessed electronically and had to be located by the prescriber. This was sometimes difficult to achieve without some understanding of the architecture of the information technology (IT) systems that were used in each site.

We searched for [a guideline] everywhere … couldn’t find it anywhere. The nurses told us what we should do, and someone on the nightshift printed off the summary page and stuck it to the wall in our office. (Interview 15, Junior Doctor.)

Guidelines also directed the referral of ambiguous orders ‘ … to the prescriber … ‘ For example, two experienced registrars in CH2 reflected on such ‘escalations’ as methods of passing a problem from one person with a limited experience of the situation to another with a similarly low base.

‘Doctor Informed’ … that’s a great one to read in the notes … It’s just a way of passing the buck really …  (Fieldnote, Observation discussion, Doctor, CH2)

These observed escalations would often see the receiving doctor ask the escalating nurse;

Okay, and what do you want me to do? (Fieldnote, Observation, CH2)

#### Knowledge and experience

There was also often an inconsistency between guidelines and actual practice. This was often complicated by the expectations and practice of parents and children themselves, who had their own adaptations and adjustments for administering medicines.

So we’re on this injection […] but its really fiddly and his blood levels were never right. Then we got chatting to someone else who’s on it, and they showed us how to dilute it […] they said ‘It’s not how we’re supposed to do it, but it works … ‘ (Interview 17, Parent)

Furthermore, acute changes in a child’s physiological status that required an adjustment in medication choice or use were often undetected by the medical team, and were identified by others, particularly pharmacists.

So we had this patient who was experiencing an adverse drug reaction to one of their medicines, and this went on for three days and we didn’t realise it was drug induced until the pharmacist came along on the Monday and told us … (Interview 9, Consultant)

In periods of uncertainty and without suitable expertise immediately available, clinicians would ‘muddle through’ to decide. A practitioner in GH1 explained that a tertiary hospital recommended the commencement of high dose methylprednisolone for the management of inflammation, but none of the local or national references referred to a suitable dose. In one reference guide, the dose was stated as ‘Consult specialist centres … ‘

… so you just google guidelines for (another disease) and … if it looks legit […] you just go ahead and refer to that. (Interview 4, Junior Doctor)

… sometimes you just need to phone a friend … (Fieldnotes, Observation discussion, Consultant, GH1)

There was a clear expectation that the most junior members of the medical and pharmacy team would undertake the majority of the medical work, reflective of the rotational nature of medical and pharmacy training in the UK.

… we have a really junior workforce … they need a lot of handholding and support … (Interview 7, Consultant)

This was my second ward … a bit more complex, which has let me get my teeth into more things. (Interview 6, Pharmacist)

Sometimes you get the feeling that some people don’t know what they’re doing … (Interview 17, Parent*)*

This relatively low level of experience, and the absence of a predictable pharmacy service created problems in maintaining patient safety. Pharmacists did not become involved with a patient’s care until after the initial administration of the medication. Consequently, pharmacist activity seldom *prevented* an adverse event.

Conversely, the nursing cohort were long-serving and experienced. Many parents of children with long term conditions were also experienced and knowledgeable of the management of their child’s condition.

### Performance influencing factors

Through this study it has been possible to observe and identify performance influencing factors in the system, both that contribute to potential ADEs and that prevent them.

#### Parents and carers

Perhaps the most obvious one is the use of parents and carers in the field. While not being a formalised part of the system from an organisational perspective (indeed, one site had no underpinning framework of standards to support parent administration of medicines), staff were happy to co-opt parents to support medication administration and co-ordinate information exchange. These parents then alleviated some of the work pressure experienced by clinical staff and retained some of their autonomy and agency while in hospital. There was an example of a child admitted on warfarin to GH1, where no one had the experience or knowledge to manage that medication, and the parent supplied full therapeutic records and undertook all monitoring and testing. In CH1 a parent was able to directly liase with another care team on behalf of the general paediatricians to answer some of their questions. Yet there was no clear expectation made of parents while they were in hospital, so they found themselves often running against the flow of hospital attitudes and assumptions. The only time parents or patients were able to access and challenge healthcare professionals was during ward rounds, or while those professionals were undertaking patient care tasks. These were often unpredictable or inconvenient for parents.

… sometimes you really need a shower, or the toilet but you know the ward round’s going on … (Interview 15, Parent)

#### Primed checking

There was a requirement for two registered nurses to ‘sign for’ medication administration and so primed checking had emerged as a way of operationally satisfying that requirement. During the observations it was clear that this process was performative at best, and there was a suggestion during the interviews that independent second checking was a policy that satisfied organisational needs for control rather than individual professional concerns.

There’s not a law in the land that requires two registered nurses to check oral medicines, but we’ve asked the children’s service if they want to remove that requirement and they’ve just kneejerked and gone ‘No!’ (Interview 14, Nurse)

Primed checks were undertaken because of a lack of people and time to undertake them, but also some nurses perceived that for many medicines the ‘independent’ second check was superfluous because the medicines were familiar, as were the patients and they were undertaking repetitive checks on the same thing.

The dose hasn’t changed in the last three shifts I’ve been giving it so why do I need to go through the whole process each time … it was right on Wednesday, it’s still right today … (Fieldnote, Observation discussion, Nurse, CH1)

#### The physical separation of teams and tasks

There was a clear lack of robust teamwork exemplified by the disparate teams (medical, nursing, pharmacy and families) working to unaligned priorities and objectives. This was reinforced by the physical separation between the groups in the ward area. Teams would come together to resolve problems as they emerged, rather than work together at the outset to mitigate their evolution. Medical staff would ask around for advice on what to do if they encountered something they were unfamiliar with.

… I’ll think if I’ve seen something similar before. I’ll ask my colleagues … and if that fails then we’ll ask the nurses … or then we’ll go to Google. (Interview 6, Junior Doctor.)

Because of time and resource constraints, medication histories on admission were brief, undetailed, and focussed only on those medicines that were important at that moment in time. This focus on the immediate aspect of the patient’s care was the norm in paediatric care. The firefighting nature of care provision in the NHS at this time meant that things were done minimally, and then the detail fleshed out later. Indeed, it was often the pharmacy service that revealed ADEs where they didn’t apply to the immediate problem.

… it was the pharmacist that identified that the [adverse] reaction we were seeing was probably related to the doxycycline. (Interview 9, Consultant)

Ironically, this disparate approach to service delivery and reliance on those with less experience contributes to distractions and interruptions which professionals are so clear are a leading cause of adverse drug events. The lack of experience of the medical and pharmacy service meant that there was an almost constant stream of enquiries and requests for help.

A nurse asks a doctor to write a discharge letter. The doctor asks ‘Can you tell me which programme I need to use for a discharge?’ (Fieldnote, Ward observation, CH1)

Many interventions were reported to mitigate these interruptions, but none were seen to be in regular use. They were all administrative controls – usually a visual signal that someone was busy and not to be disrupted – but they were not used because they did not fit with workflows. In CH1 managers and MSOs advocated for the use of red plastic aprons, but on observation days the boxes of these were usually found propping up a laptop on a trolley. In another site, a similar intervention imposed by the ward team themselves had simply not been ordered for some time.

#### Technology to reduce ADEs

During this study several complex technologies were observed that attempted to improve medicines safety. The best example of this is the introduction of barcode medicines administration (BCMA) system, whereby the scanning of barcodes on products and patient identification wristbands verified the right patient, the right drug and the right time. The organisational driver for this was to reduce medication administration errors, by enforcing the second checking process described above. However, there were several physical limitations to this process that made using it in the clinical setting difficult.

First there were different patient identification systems within the hospital. This meant that identification wristbands had to be changed on admission to the ward to ensure the correct identification was available. However, this was dependent on printers being available and functional, which was often observed to be a problem.

Honestly, sometimes I feel like IT support with these bloody printers … ! (Fieldnotes, ward observation, CH1)

Second, the equipment itself was deficient. It was based on laptop computers that had to be wheeled from room to room and were often sequestered for other tasks or in use by other people for other purposes. And third, many medications in the inventory had unreadable barcodes. The use of oral liquid medicines led to inevitable contamination of labels rendering barcodes illegible, and medicines that parents brought in from home were also unreadable. Nursing staff consequently did not engage with the digital system, preferring to use manual checking processes instead:

So if I [mess] around with the barcode, it’s going to delay patient care, so I’ll override it and manually sign it off. (Observation, CH1)

Despite repeated intervention at an organisational level to encourage increased adherence to BCMA standards, there were concerns that the drive for the BCMA was more managerial than clinical.

Oh yeah … if it’s not doing what it’s supposed to then it should just be binned, but some of the ward managers … like to have the data from it … (Fieldnotes, Pharmacy Manager discussion, CH1)

## Discussion

To our knowledge this is the first multi-site qualitative study of paediatric medicines safety practices using robust theoretical principles from ergonomics and human factors – namely, sociotechnical theory (Carayon and Smith 2000). This paper builds on and extends earlier explorations of medicines safety for children and young people by using ethnographic observations and interviews with participants engaged in the practice of medication safety processes as ‘work-as-done.’(Sutherland, Phipps, et al. 2019) Wong et al. identified that issues with prescribing may be related to education and communication issues among prescribers and other healthcare providers (Wong, Wong, and Cranswick 2009). Sutherland et al. also identified the cognitive burden associated with prescribing and managing medicines for children and young people in paediatric critical care (Sutherland, Phipps, et al. 2019). Our study builds on these studies by examining the interrelationship between the clinical environment and everyday informal safety practice, with a key finding being that the way services and the wards are structured as physical environments creates barriers to effective inter-professional communication across paediatric teams.

We have also identified with real-world observational data that the challenges and barriers to patient safety identified by Hignett et al. in 2015 persist (Hignett et al. 2018). In their quantitative survey study of healthcare staff they identified resource issues, systems design deficiencies and sub-optimal teamworking as barriers to patient safety. In this ethnographic study we have shown that within English paediatric in-patient units, staff work in ‘silos’ with poor communication pathways, using inaccessible guidelines and procedures, and have to manage complex and unstandardised work processes. Many interventions implemented by healthcare organisations to protect against medication error specifically have taken a human-centric, behavioural approach without acknowledging the difficult organisational and cultural landscape in which these people work (Catchpole 2013). It is therefore not surprising that these interventions have not been well received by workers, or been effective.

Consequently, we have identified the importance of informal medication safety practice. Staff regularly ‘muddle through’, adapting and adjusting their working practices. This involved them drawing on multiple sources of knowledge beyond formal guidelines to address the complexity of everyday clinical practice and keep processes on track (Gabbay and May 2004). There was also evidence of trade-offs being undertaken by healthcare staff to manage their day to day work in response to unexpected events, or situations that create tension in normal decision making (Sujan, 2021). Both these phenomena illustrate the considerable resilience within the medication systems for children and young people in hospital that by and large workers, patients and their families are able to adapt and work together to resolve problems where individuals may not be able to resolve matters alone. We argue that these adaptive processes are impossible to detect using traditional epidemiological techniques, nor through interview or focus group methods, because this would yield only ‘work as disclosed’. (Shorrock 2022) We have also identified the important role that parents often play as part of the system but this was not the focus of this paper, and merits separate in-depth exploration.

While there are legitimate concerns around the use of unlicensed and off-label medicines in children and young people (Standing and Tuleu 2005; Rocchi et al. 2010; Bellis, Kirkham, and Pirmohamed 2013; Bellis, Kirkham, and Pirmohamed 2013), this study has reported many episodes where off-label medication administration was chosen despite the availability of a licenced medicine. This trade-off likely emerged as an unintended consequence of a focus on missed and omitted doses within the NHS, and thus sheds light on a wider problem with NHS safety policy that focusses on specific problems, without considering their wider systemic impacts (National Patient Safety Agency, 2010). As we have explored in this paper, much inference is made of cause-and-effect using abstract epidemiological data without exploration of the complexities of diverse systems. Yet the example of off-label administration illustrates the argument made by Rasmussen’s risk management model that local actions are performed to serve (and so are shaped by) higher-level system functions (Rasmussen 1997). Further, the interruption and distraction-rich nature of the modern ward environment meant that there was seldom a safe cognitive space in which to conduct complex processes thus there is a clear suggestion in this study that healthcare workers will choose the nearest available option to ensure their goals are met.

Additionally, this study has exposed a critical weakness in the medicines management systems and processes to protect children and young people from medication associated harm. At a systemic level the only operational control in place to prevent harmful medication error was the independent second check on administration but these were only intended to be undertaken by nurses who had insufficient time to complete them. Observed second checks in this study were largely performative, with the administering nurse priming the checking nurse through the elements that required verification. In the wider literature the evidence base for the effectiveness of second checks as a preventative mechanism for medication error is equivocal with no robust reduction in harmful medication administration errors where they are implemented (Armitage 2008; Alsulami, Choonara, and Conroy 2013; Koyama et al. 2020). Our study now provides insights into why these may be so challenging to implement, and it comes down to a lack of time, a lack of co-ordination of medication related tasks, and risk-perceptions of the checking nurses.

The way that healthcare professionals worked collaboratively could be described as examples of ‘boundary work’, described by Allen whereby nursing staff use ‘atrocity stories’ to delineate their actions from those of others, and to reinforce their own professional roles and responsibilities (Dingwall 1977; Allen 2001). This was exemplified through the co-option of parents and their knowledge and expertise medication, to support the work of medical and nursing staff, which runs counter to the formalised hierarchies in modern healthcare organisations (Freidson 1970). This was also demonstrated at an organisational level where the ‘prescriber’ was the point of reference for all medication-related problems, but in reality it was the nursing staff who were the final arbiters of what constituted ‘safe’ medication administration. This was a responsibility taken seriously by nursing staff because they carried most of the accountability for the administration of medicines. Thus there are flexible boundaries within the care of hospitalised children, with nursing staff having greater clinical knowledge and experience than those enshrined with that experience by their job titles (Nancarrow and Borthwick 2005). This study has also provided new insights into how this knowledge is obtained, and we have identified that parents and families share medication-related information and experience with nursing staff and also advocate for their children while they are in hospital, but this advocacy may not be formally acknowledged.

What has been revealed by this study is that teamwork in this setting is more fluid. This may have led to poor collective understanding of the priorities, objectives and working practices of the different actors in the system – medical, nursing, pharmacy and family. Our recent study of the purposes and functions of the medication safety system in this setting using Work Domain Analysis offers much deeper insights into the diversity of tasks and tools, and the difficulties in aligning these across multiple actors (Sutherland et al. 2023). Further, while being an integral part of the system families sit outside of organisational control, but have autonomy and agency over their care, and offer crucial information and practical expertise around administration of medicines to their children. Professional communication was transactional, and professionals only came together briefly to resolve problems together and would then separate and go back to their previous tasks. This would suggest that there is an absent mental model from the ‘team’ in these settings, because there is no team (Rouse, Cannon-Bowers, and Salas 1992). Much study of mental models has been centred on teams that function for relatively long periods of time, or are stable (e.g. in Intensive Care Units or Emergency Departments) but there is an issue here where organisational and physical structures in place to support patient flow through the hospital actually impedes good teamwork (Singer et al. 2009; Burtscher and Manser 2012). This lack of shared appreciation of each other’s roles and objectives may go some way to explain why technical interventions to mitigate interruptions were ineffective. Distractions and interruptions are likely associated with social and relational problems that cannot be ‘solved’ with simple technical ‘fixes’ such as aprons and laminated signage.

The stories that our participants told through this study serve as ‘cautionary tales’ of how adverse events emerge; it would appear that certain expectations of safety practices laid upon nursing staff are incompatible with work in busy and unpredictable systems. This notion that safety expectations and organisational interventions are undeliverable has also been explored in a large ethnographic study from the United States (Hawkins and Morse 2022). Thus we conclude that the culture of independent second checks is impossible to deliver in the current climate and unsupported by empirical evidence. We also contend that this is not just the case for administration checks. Pharmacy professionals expended a substantial proportion of their time on the conduct of medicines reconciliations. Again, these were driven by national priorities, but reflecting on the empirical literature, medicines reconciliation reduces potential avoidable harm only in those children with medical complexity, or those who are on five or more concomitant medicines (Coffey et al. 2009; Stone et al. 2010; Huynh et al. 2012; Terry et al. 2010).

This study is not without its potential limitations. We must acknowledge that the observational and interview data were collected by a single researcher, which will inevitably reduce the richness that other perspectives may have brought to the data collection. To account for this limitation, we ensured that the analysis was conducted using a broad team of methodological experts and those with lived experience. The data produced from this study is also broad and rich and other insights into the systems and their interactions are possible, but this was not feasible within the boundaries of a doctoral research project and the broad question relating to potential systemic contributory factors to drug-related problems. There may also be impacts related to service adaptations related to the COVID-19 pandemic and how those may affect our findings, however many of the interventions implemented during this study (social distancing, mask wearing, reduced visiting) are still in effect in English hospitals so our findings remain valid.

## Conclusions

Medicines safety is a complex socio-technical activity enacted through four discrete groups of people – doctors, nurses, pharmacy staff and families. A combination of the physical environment, traditional work systems and team norms contribute collectively to systemic and structural barriers to medicines safety. The physical environment isolates these groups and obstructs effective teamwork. Consequently, there is no shared mental model of medicines safety work and actors within the system have only limited understanding of roles, responsibilities and limitations of others. This is exacerbated by operational objectives being limited to individual professions and not aligned with other professionals. Professionals share information and concerns in a transactional rather than collaborative manner. Notwithstanding these structural and communication issues, problem solving and dealing with uncertainty is dealt with through shared experience and memory, and actors ‘muddle through’ using the combined experience and knowledge of everyone around them, rather than formal policies and guidelines. Many interventions to support medicines safety are incompatible with day-to-day work, and consequently are poorly adhered to.

This ethnographic study has demonstrated that formal interventions to support medicines safety have not been designed or developed with informal work routines and practices in mind. Similarly, medication safety is not incorporated in the design of hospital buildings. The result is adaptations to manage the resulting difficulty in accessing and administering medicines; that is, to provide resilience within the system. Further consideration should therefore be given to these specific adaptions in the paediatric context, as well as how we build on them to improve safety. A sociotechnical analysis of paediatric work systems (using, for example, cognitive work analysis) could support future redesign of these systems, particularly around resource allocation and use, and understanding of the strategies that may be available to manage work.

## Supplementary Material

Supplemental Material

## Data Availability

The data that support the findings of this study are available via Figshare at the following URL: https://doi.org/10.48420/24925329.v1

## Abbreviaxtions

ADE: Adverse Drug Event
MSO: Medicines Safety Officer
WoW: Workstation on Wheels
