# Supplementary material for: Understanding the informal aspects of medication processes to maintain patient safety in hospitals: a sociotechnical ethnographic study in paediatric units
Source: Ergonomics. 2024 Apr 1;68(3):444–58. doi: 10.1080/00140139.2024.2333396 (PMC11835306; doi:10.1080/00140139.2024.2333396)
Supplement: Supplemental Material [file TERG_A_2333396_SM8508.pdf]

MANCHESTER
1824
The University of Manchester

Medicines Optimisation in Paediatric In-patients (MOPPEt)

Professionals interview schedule

Aims & Objectives

- Develop deep understanding of medication systems
- Explore medication practices from the perspective of the operator

Introduction

1. Introduce myself
2. Explain the nature of the research
3. Stress confidentiality
4. Explain how the data generated will be used – transcription, reporting and anonymization
5. Introduce the recorder
6. Offer the participant opportunity to ask questions

QUESTIONS

1. Tell me about your experience of medication systems in your work.

2. What do you perceive the objectives of the medication systems in your work are?
    a. *How do you know whether they're being met*
    b. *What do you need to do to meet those objectives*
    c. *How is the work done?*
    d. *Who and what is involved?*

3. Now I want to talk about Drug Related Problems – these can be adverse drug reactions, medication errors or adverse drug events
    a. *What sort of DRPs have you encountered and how do you identify them?*
    b. *How do you resolve them?*
    c. *What information do you give to parents and carers*

4. Now think of a situation where you've been involved with a DRP:
    a. *What was happening around you*
    b. *What were you expecting to happen*
    c. *What were your options*
    d. *What did you choose to do*
    e. *Why?*
    f. *What happened?*
    g. *What happened to you?*
